# Associations Between Hypertension, Angiotensin-Converting Enzyme Inhibitors, and Physical Performance in Very Old Adults: Results from the ilSIRENTE Study

**DOI:** 10.14283/jfa.2024.15

**Published:** 2024-03-06

**Authors:** Helio José Coelho-Junior, R. Calvani, M. Tosato, A. Álvarez-Bustos, F. Landi, A. Picca, Emanuele Marzetti

**Affiliations:** 1 Department of Geriatrics, Orthopedics and Rheumatology, Università Cattolica del Sacro Cuore, L.go F. Vito 1, 00168, Rome, Italy; 2 Fondazione Policlinico Universitario “A. Gemelli” IRCCS, Largo A. Gemelli 8, 00168, Rome, Italy; 3 Biomedical Research Center Network for Frailty and Healthy Ageing (CIBERFES), Institute of Health Carlos III, 28029, Madrid, Spain; 4 Department of Medicine and Surgery, LUM University, Str. Statale 100 km 18, 70100, Casamassima, Italy

**Keywords:** Cardiovascular disease, antihypertensive drugs, physical function, sarcopenia, elderly

## Abstract

**Background:**

Results regarding the associations between hypertension-related parameters and physical performance in older adults are conflicting. A possible explanation for these divergent results is that investigations may not have adjusted their analyses according to the use of angiotensin-converting enzyme inhibitors (ACEIs).

**Objectives:**

To examine the associations between hypertension-related parameters, ACEI use, and a set of physical performance tests in very old adults.

**Design:**

Cross-sectional study from the ilSIRENTE database.

**Setting:**

Mountain community of the Sirente geographic area (L'Aquila, Abruzzo, Italy).

**Participants:**

All persons born in the Sirente area (13 municipalities) before 1 January 1924 and living in that region at the time of study were identified and invited to participate. The final sample included 364 older adults (mean age: 85.8 ± standard deviation [SD] 4.8).

**Measurements:**

Physical performance was assessed using isometric handgrip strength (IHG), walking speed (WS) at normal and fast pace, 5-time sit-to-stand test (5STS), and muscle power measures. Blood pressure (BP) was measured after 20 to 40 min of rest, while participants sat in an upright position. Drugs were coded according to the Anatomical Therapeutic and Chemical codes. ACEIs were categorized in centrally (ACEI-c) and peripherally (ACEI-p) acting. Blood inflammatory markers, free insulin-like growth factor 1 (IGF-1), and IGF-binding protein 3 (IGFBP-3) were assayed.

**Results:**

Results indicated that 5STS test was significantly and negatively associated with diastolic BP values. However, significance was lost when results were adjusted for ACEI use. Participants on ACEIs were more likely to have greater specific muscle power and higher blood levels of IGFBP-3 than non-ACEI users. When participants were categorized according to ACEI subtypes, those on ACEI-p had higher blood IGF-1 levels compared with ACEI-c users.

**Conclusions:**

The main findings of the present study indicate that ACEI use might influence the association between hypertension-related parameters and neuromuscular parameters in very old adults. Such results may possibly be linked to the effects of ACEI-p on the IGF-1 pathway.

## Introduction

**T**he aging process is associated with molecular and cellular changes that modify the function of physiological systems and predispose to the development of chronic conditions (1). The prevalence of arterial hypertension, a condition characterized by sustained elevations in blood pressure (BP), increases with age, affecting more than 70% of those 65 and older (2). If not adequately treated, hypertension increases the risk of numerous negative health events (3). Declining physical performance is a major health concern in older adults (4). Physical function starts to deteriorate early in life, approximately during the third decade, and drops dramatically in advanced age (5). Like hypertension, low physical performance predisposes to the occurrence of negative health outcomes (6).

Hypertension and poor physical function share numerous pathophysiological mechanisms, which led some authors to suggest that these conditions could occur simultaneously and be associated. However, studies found conflicting results with investigations reporting positive (7), inverse (8, 9), and null associations (10, 11). A possible explanation for these divergent findings is that some studies did not adjust the analyses according to the use of angiotensin-converting enzyme inhibitors (ACEIs).

ACEIs are a class of widely used antihypertensive drugs (3). ACEIs significantly reduce the risk of myocardial infarction as well as cardiovascular and all-cause mortality (12). Furthermore, ACEIs might counteract age-related decline in neuromuscular function (13), possibly by blunting the negative effects of angiotensin II (ANGII) on skeletal muscle mass and function (14, 15, 16). ANGII-induced muscle atrophy is associated with reduced local and systemic insulin-like growth factor 1 (IGF-1) and insulin-like growth factor binding protein 3 (IGFBP3) levels (16, 17, 18, 19). Treatment with ACEIs may increase the expression of IGF-1 and its downstream proteins (e.g., AKT) (20, 21, 22). These adaptations might contribute to the maintenance of neuromuscular function, given that IGF-1 is a major regulator of muscle protein synthesis (41, 42, 43).

However, other studies did not find significant associations between ACEI use and better neuromuscular performance (23, 24, 25). The lack of examination of centrally (ACEI-c) and peripherally (ACEI-p) acting agents might explain, at least partly, these conflicting results (26).

To expand the knowledge on the subject, the present study was conducted to examine the associations between hypertension-related parameters and a set of physical performance tests in a well-characterized cohort of very old adults. The possible mediator role of ACEIs and their subtypes in this relationship was also analyzed.

## Material and Methods

Data for the present investigation were obtained from the IlSIRENTE study database (27). IlSIRENTE was a prospective cohort study conducted in the mountain community of the Sirente geographic area (L'Aquila, Abruzzo) in Central Italy. The study aimed to characterize health and functional trajectories in a homogeneous sample of very old adults through the collection of detailed clinical and biological information (27). IlSIRENTE was compliant with the principles of the Declaration of Helsinki and the protocol was approved by the Ethics Committee of the Università Cattolica del Sacro Cuore (Rome, Italy). All participants signed an informed consent prior to enrolment.

### Study population

A list of all persons living in the Sirente area was obtained in October 2003 from the registry offices of the 13 municipalities involved in the study. Potential study participants were subsequently identified by selecting all those born before 1 January 1924 and currently living in that region. The total sample enrolled in the ilSIRENTE study consisted of 364 older adults.

### Data collection

Baseline assessments began in December 2003 and were completed in September 2004. Follow-up visits took place after 24 months of baseline assessment. Information on medical history, medications, and lifestyle habits (e.g., smoking, alcohol consumption, physical activity) was collected using validated questionnaires (27). Body height and weight were measured through a stadiometer and an analog medical scale, respectively. The body mass index (BMI) was then calculated as the ratio between body weight (kg) and the square of height (m^2^). Calf circumference was taken on the dominant leg by measuring the largest girth (cm) between ankle and knee joints using an anthropometric tape while the participant was in a seated position. Values were rounded to nearest 0.1 cm. Appendicular skeletal muscle (ASM) was estimated based on the equation developed by the COCONUT Study Group (28):

**Figure:**
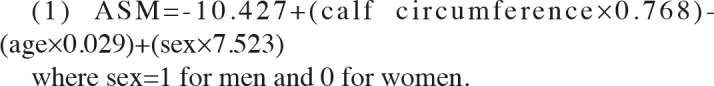

Isometric handgrip (IHG) strength was measured in both hands. The test was performed with participants sitting comfortably on a chair with their shoulders in a neutral position. The arm being assessed had the elbow flexed at 90° near the torso, and the hand neutral with thumb up. A maximal contraction was performed over four seconds using a handheld hydraulic dynamometer (North Coast Hydraulic Hand Dynamometer; North Coast Medical, Inc., Morgan Hill, CA, USA). The highest reading (kg) was used for the analysis. Walking speed (WS) was evaluated by measuring the participant's usual and fast WS (in m/s) over a 4-m course. For the 5-time sit-to-stand (5STS) test, participants were requested to stand up and sit down from a chair with their arms folded across the chest five times as quickly as possible. The time needed to complete the task was recorded in seconds from the moment participants raised their buttocks off the chair to the instant they seated at the end of the fifth stand. Absolute muscle power values were calculated according to the equation proposed by Alcazar et al (29, 30):

**Figure:**
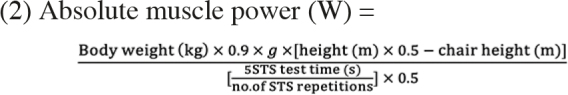

Relative (adjusted by body weight), allometric (adjusted by height squared), and specific (adjusted by ASM) muscle power values were subsequently calculated as follows:

**Figure:**
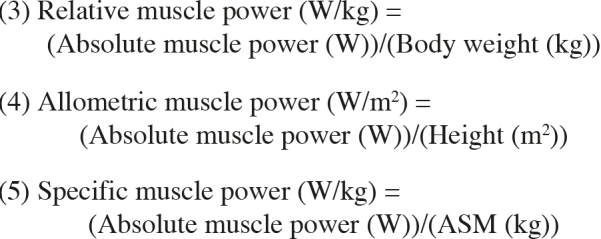

BP was measured after 20 to 40 min of rest, while participants sat in an upright position. Three measurements were taken from the left arm, and one from the right arm. BP values were then calculated from the average of the last two measures of the left arm plus the measure obtained from the right arm. Four hypertension groups were identified according to: a) self-report of physician diagnosis (CLI-HTN), b) high systolic BP (i.e., ≥130 mmHg) (SBP-HTN), c) high diastolic BP (i.e., ≥80 mmHg) (DBP-HTN), and d) high systolic and diastolic BP (BP-HTN). Drugs were coded according to the Anatomical Therapeutic and Chemical codes. Analytical variables were created for the use of ACEIs and other antihypertensive drugs. ACEIs were categorized in ACEI-c (i.e., captopril, lisinopril, perindopril, fosinopril, trandolapril, zofenopril, and ramipril) and ACEI-p (i.e., enalapril, quinapril, benazepril, and moexipril).

Blood samples were obtained from approximately 97% of participants. Blood was drawn after overnight fasting by a trained phlebotomist according to a standardized protocol. Blood samples were immediately centrifuged at 4 °C and stored at −80 °C until analysis. Standard determinations of glucose, calcium, sodium, potassium, magnesium triglycerides, low-density lipoprotein (LDL), high-density lipoprotein (HDL), and total cholesterol were performed on an Olympus 2700 instrumentation (Olympus, Italy). C-reactive protein (CRP), interleukin-6 (IL-6), and tumor necrosis factor-α (TNFα) were measured in plasma by enzyme-linked immunosorbent assays (ELISA; High Sensitivity Quantikine KitR&D Systems, Minneapolis, MN). Plasma concentrations of free IGF-1 and IGF binding protein 3 (IGFBP3) were measured by radioimmunoassay (Diagnostic Systems Laboratories, USA; obtained from Pantec S.r.l., Turin, Italy). All assays were run in duplicate, and the average values were used for the analyses.

### Statistical analysis

Continuous variables are expressed as mean ± standard deviation (SD), while categorical variables are reported as absolute numbers and percentages. Data were not normally distributed. However, non-Gaussian distribution may be ignored if large sample sizes (>30–40) participants) with values representative of a “real population” are analyzed (31, 32). Comparisons between groups with and without hypertension, ACEIs and non-ACEIs, and ACEI-c and ACEI-p were performed using independent t-tests. Pearson's correlation was used to explore the relationship between BP and performance on physical tests. Linear regressions were conducted to test the association between hypertension-related parameters and physical performance tests. The final models were adjusted for age, sex, BMI, physical activity levels, any cardiovascular diagnosis, smoking status, unintentional loss of weight, systolic BP, diastolic BP, and ACEI use. For all tests, the level of significance was set at 5% (p < 0.05). All p values were determined by two-tailed tests. Analyses were performed using the SPSS software (version 23.0, SPSS Inc., Chicago, IL, USA).

## Results

### Characteristics of study participants

Table 1 shows the main characteristics of study participants according to their hypertensive status. Compared with participants with no hypertension, higher diastolic BP values were observed in SBP-HTN, DBP-HTN, and BP-HTN groups, but not in those with CLI-HTN. When participants were categorized according to the clinical diagnosis of HTN, those with hypertension had higher blood glucose and calcium levels than their normotensive counterparts. Participants classified as hypertensive according to BP levels were younger and had slightly greater BMI values. DBP-HTN and BP-HTN groups showed a dyslipidemic profile, characterized by higher total blood cholesterol, LDL- and HDL-cholesterol, and triglycerides levels. IHG and ASM were significantly greater in both DBP-HTN and BP-HTN groups.

**Table 1:** Main characteristics of study participants (n=364)

Variables	CLI-HTN		SBP-HTN		DBP-HTN		BP-HTN	
	Yes (n= 185)	No (n= 179)	Yes (n=251)	No (n=113)	Yes (n= 245)	No (n= 119)	Yes (n= 212)	No (n= 80)
Age (years)	85.5 ± 4.7	86.1 ± 4.9	85.4 ± 4.4	86.9 ± 5.6*	85.0 ± 4.2	87.4 ± 5.5*	85.0 ± 4.1	87.6 ± 5.8*
Women (n, %)	128 (69.2)	116 (64.8)	166 (66.1)	78 (69.0)	165 (67.3)	79 (66.4)	144 (67.9)	57 (71.3)
BMI (kg/m^2^)	25.7 ± 4.5	25.4 ± 4.5	25.9 ± 4.2	24.9 ± 5.0*	26.3 ± 4.5	24.0 ± 4.2*	26.1 ± 4.2	23.8 ± 4.3*
Weight (kg)	63.0 ± 12.4	62.3 ± 13.2	63.3 ± 12.1	60.6 ± 14.0	64.4 ± 12.7	58.9 ± 12.2*	63.8 ± 12.1	57.1 ± 11.7*
Height (m)	1.56 ± 0.09	1.56 ± 0.09	1.56 ± 0.09	1.55 ± 0.09	1.56 ± 0.09	1.56 ± 0.09	1.56 ± 0.09	1.54 ± 0.09
ASM (kg)	14.9 ± 5.1	15.2 ± 5.4	15.4 ± 5.0	14.2 ± 5.8	15.5 ± 5.0	14.1 ± 5.7*	15.4 ± 4.8	13.3 ± 5.7*
IHG strength (kg)	28.3 ± 14.5	27.4 ± 14.9	29.6 ± 14.1	24.0 ± 15.1	29.9 ± 13.9	23.7 ± 15.2*	29.8 ± 13.7	21.2 ± 14.1*
WS at normal pace (m/s)	0.56 ± 0.25	0.57 ± 0.24	0.57 ± 0.24	0.54 ± 0.27	0.58 ± 0.25	0.53 ± 0.24	0.57 ± 0.24	0.49 ± 0.24*
WS at fast pace (m/s)	0.72 ± 0.34	0.73 ± 0.34	0.74 ± 0.32	0.67 ± 0.38	0.75 ± 0.33	0.67 ± 0.37	0.74 ± 0.32	0.60 ± 0.37*
5STS (s)	15.2 ± 6.1	15.1 ± 7.7	15.3 ± 7.1	14.8 ± 6.4	14.8 ± 6.8	16.1 ± 7.2	15.1 ± 7.2	16.1 ± 7.5
Muscle power								
Absolute (W)	139.3 ± 59.3	143.2 ± 65.0	140.6 ± 58.4	143.1 ± 72.7	145.0 ± 59.5	131.0 ± 68.1	141.6 ± 59.3	127.5 ± 77.7
Relative (W/kg)	2.1 ± 0.7	2.2 ± 1.0	2.1 ± 0.7	2.2 ± 1.2	2.1 ± 0.7	2.1 ± 1.2	2.1 ± 0.7	2.1 ± 1.5
Allometric (W/m^2^)	55.4 ± 19.4	57.1 ± 25.7	55.9 ± 19.4	57.4 ± 30.8	57.6 ± 19.6	52.5 ± 29.6	56.4 ± 19.7	52.3 ± 36.3
Specific (W/kg)	2.4 ± 0.29	2.4 ± 0.26	2.4 ± 0.27	2.4 ± 0.28	2.4 ± 0.27	2.4 ± 0.28	2.4 ± 0.27	2.4 ± 0.27
Current smoking (n, %)	40 (21.6)	44 (24.6)	59 (23.5)	25 (22.1)	56 (22.9)	28 (23.5)	49 (23.1)	18 (22.5)
Loss of weight (n, %)	36 (19.5)	33 (18.4)	44 (17.5)	25 (22.1)	42 (17.1)	27 (22.7)	37 (17.5)	20 (25.0)
Multimorbidity (n, %)	2 (1.1)	9 (4.9)*	6 (2.4)	5 (4.4)	6 (2.4)	5 (4.2)	5 (2.4)	4 (5.0)
Physically active (n, %)	33 (17.8)	32 (17.9)	52 (20.7)	13 (11.5)	49 (20.0)	16 (13.4)*	45 (21.2)	9 (11.3)*
SBP (mmHg)	148.4 ± 26.0	141.8 ± 23.1*	157.2 ± 19.0	117.9 ± 10.9*	154.1 ± 22.2	126.3 ± 18.9*	159.0 ± 19.7	115.8 ± 11.4*
DBP (mmHg)	81.6 ± 14.5	80.0 ± 11.6	85.2 ± 12.5	71.1 ± 8.6*	87.1 ± 11.0	68.0 ± 6.1*	88.0 ± 11.5	66.9 ± 6.5*
ACEI (%)	103 (55.6)	31 (17.3)	100 (39.8)	34 (30.0)	91 (37.1)	43 (36.1)	112 (52.8)	22 (27.5)
Calcium channel blocker (%)	45 (24.3)	16 (9.0)	41 (16.3)	20 (17.6)	37 (15.1)	24 (20.1)	39 (18.3)	24 (30.0)
Hypertension (%)	185 (100)	0 (0.0)	137 (1.8)	48 (42.4)	128 (52.2)	57 (47.8)	115 (54.2)	53 (66.2)
Diabetes mellitus (%)	26 (14.0)	9 (5.0)	29 (11.5)	6 (5.3)	27 (11.0)	8 (6.7)	26 (12.2)	8 (10.0)
Stroke (%)	10 (5.0)	7 (4.0)	8 (3.1)	7 (6.1)	7 (2.8)	10 (8.4)	5 (2.3)	3 (3.75)
CKD (%)	2 (1.0)	1 (0.5)	2 (0.7)	1 (0.8)	2 (0.8)	1 (0.8)	2 (0.9)	1 (1.25)
Heart failure (%)	13 (7.0)	8 (4.4)	13 (5.1)	9 (8.0)	14 (5.7)	8 (6.7)	12 (5.6)	10 (12.5)
Glucose (mg/dL)	123.1 ± 46.2	113.2 ± 36.5*	118.6 ± 42.1	117.5 ± 41.8	118.5 ± 41.2	118.0 ± 35.6	118.6 ± 42.2	117.4 ± 44.7
Total cholesterol (mg/dL)	197.8 ± 46.3	195.2 ± 43.3	200.7 ± 44.8	187.3 ± 43.5	202.3 ± 44.0	184.4 ± 44.3*	202.8 ± 44.1	182.3 ± 42.9*
LDL-cholesterol (mg/dL)	128.5 ± 38.8	131.6 ± 38.2	133.0 ± 39.1	123.5 ± 36.4	135.4 ± 38.7	118.9 ± 35.6*	135.4 ± 38.4	118.4 ± 33.2*
HDL-cholesterol (mg/dL)	46.4 ± 12.6	44.8 ± 14.2	46.5 ± 14.2	43.5 ± 11.3	46.6 ± 14.3	43.5 ± 11.1*	46.8 ± 14.9	42.9 ± 11.8*
Triglycerides (mg/dL)	145.0 ± 61.2	147.2 ± 63.5	148.9 ± 63.4	139.6 ± 59.5	151.4 ± 65.3	134.8 ± 53.9*	150.4 ± 65.5	131.7 ± 55.7*
Sodium (mmol/L)	138.1 ± 5.6	138.3 ± 4.7	138.4 ± 5.6	137.7 ± 4.0	138.6 ± 5.7	137.3 ± 3.7*	138.6 ± 5.9	137.4 ± 3.9
Potassium (mmol/L)	4.3 ± 0.43	4.4 ± 0.52	4.3 ± 0.42	4.3 ± 0.59	4.3 ± 0.45	4.3 ± 0.55	4.3 ± 0.43	4.3 ± 0.61
Magnesium (mg/dL)	1.96 ± 0.57	1.92 ± 0.20	1.94 ± 0.49	1.94 ± 0.22	1.93 ± 0.50	1.96 ± 0.20	1.94 ± 0.53	1.96 ± 0.21
Calcium (mg/dL)	9.0 ± 0.52	8.8 ± 0.59*	8.9 ± 0.55	8.7 ± 0.55	8.9 ± 0.55	8.7 ± 0.54*	9.0 ± 0.56	8.7 ± 0.57*


Data are shown as mean ± standard deviation and n, %. *p<0.05 Abbreviations: 5STS, 5-time sit-to-stand; ACEI, angiotensin converting enzyme inhibitors; ACEI-HTN, hypertension treated with angiotensin converting enzyme inhibitors; ASM, appendicular skeletal muscle; BP-HTN, hypertension based on high systolic and diastolic blood pressure; CLI-HTN, medical diagnosis of hypertension; DPB, diastolic blood pressure; DBP-HTN, hypertension based on high diastolic blood pressure; IHG, isometric handgrip strength; SBP, systolic blood pressure, SBP-HTN, hypertension based on high systolic blood pressure; WS, walking speed.

When CLI-HTN participants were compared according to the use of ACEIs, greater specific muscle power was observed in those on ACEIs relative to CLI-HTN participants on other antihypertensive drugs (Data now shown). No other differences were detected between groups.

### Relationship between blood pressure and physical performance

Results of correlation analysis between physical function and systolic and diastolic BP values are shown in Table 2. Systolic BP was significantly and positively associated with IHG and ASM. No other significant correlations were observed.

**Table 2:** Pearson's correlation between blood pressure and physical performance tests (n=364)

Variables	IHG strength	WS at normal pace	WS at fast pace	5STS	Absolute MP	Relative MP	Allometric MP	Specifie MP	ASM
SBP	0.178*	0.067	0.106	−0.026	0.004	−0.007	−0.015	0.033	0.132*
DBP	0.113*	0.036	0.039	−0.119	0.042	0.038	0.050	−0.020	0.035


* p < 0.05; Abbreviations: 5STS, 5-time sit to stand; ASM, appendicular skeletal muscle; DBP, diastolic blood pressure; IHG, isometric handgrip; MP, muscle power; SBP, systolic blood pressure; WS, walking speed

Results of adjusted regression analysis for the association between physical function and hypertension categories are listed in Table 3. Unadjusted analysis is shown in supplementary Table 1. In the unadjusted analysis, IHG and ASM were significantly associated with hypertension categorized according to high systolic BP and/or diastolic BP values, as well as with systolic BP as a continuous variable. A linear association was observed between IHG and diastolic BP. WS at normal and fast pace was associated with hypertension based on high systolic and diastolic BP values. Absolute, relative, and specific muscle power measures were positively associated with the use of ACEIs. After adjusting the analysis for potential confounders, 5STS was negatively and significantly associated with diastolic BP as a continuous variable. Significance was lost when results were further adjusted by ACEI use. Moreover, participants on ACEIs were more likely to have greater specific muscle power. No other significant associations were observed.

**Table 3:** Adjusted regression analysis for the association between hypertension-related parameters and physical performance

	CLI-HTN		SBP-HTN		SBP continuous		DBP-HTN		DBP continuous		BP-HTN		ACEI-HTN	
	Adjusted β (95% CI)	p	Adjusted β (95% CI)	p	Adjusted β (95% CI)	p	Adjusted β (95% CI)	p	Adjusted β (95% CI)	p	Adjusted β (95% CI)	p	Adjusted β (95% CI)	p
IHG (kg)	1.54 (0.44. −2.39)	0.105	1.54 (−2.39. 5.48)	0.442	0.04 (−0.00. 0.09)	0.105	1.47 (−2.24. 5.19)	0.435	0.04 (−0.04. 0.14)	0.303	2.65 (−2.19. 7.50)	0.282	2.54 (−1.02. 6.10)	0.161
WS at normal pace (m/s)	0.00 (−0.04. 0.05)	0.739	−0.00 (−0.08. 0.07)	0.934	0.00 (−0.00. 0.00)	0.765	0.05 (−0.02. 0.12)	0.184	0.00 (−0.00. 0.00)	0.493	0.04 (−0.05. 0.14)	0.346	0.02 (−0.04. 0.09)	0.512
WS at fast pace (m/s)	−0.00 (−0.07. 0.06)	0.915	0.00 (−0.09. 0.11)	0.867	0.00 (−0.00. 0.00)	0.329	0.03 (−0.06. 0.13)	0.464	0.00 (−0.00. 0.00)	0.541	0.04 (−0.08. 0.17)	0.485	0.04 (−0.05. 0.14)	0.373
ASM (kg)	0.57 (−1.94. 3.09)	0.655	−0.34 (−1.43. 0.73)	0.529	0.07 (−1.02. 1.16)	0.898	0.09 (−0.92. 1.11)	0.854	0.00 (−0.02. 0.02)	0.880	−0.16 (−1.49. 1.16)	0.809	−0.27 (−0.99. 0.45)	0.463
5STS (s)	−0.23 (−1.89. 1.43)	0.782	1.64 (−0.94. 4.24)	0.212	−0.01 (−0.04. 0.02)	0.480	−0.711 (−3.16. 1.73)	0.568	−0.08 (−0.14. −0.01)	0.017	0.53 (−2.81. 3.89)	0.752	−1.0 (−3.10. 1.08)	0.342
Absolute muscle power (W)	1.53 (−11.2. 14.2)	0.812	−9.91 (−29.8. 10.0)	0.328	−0.03 (−0.30. 0.23)	0.793	−2.61 (−21.4. 16.2)	0.785	0.38 (−0.11. 0.87)	0.133	−5.92 (−31.4. 19.5)	0.647	−2.33 (−16.4. 11.7)	0.744
Relative muscle power (W/kg)	0.01 (−0.18. 0.21)	0.877	−0.18 (−0.49. 0.12)	0.233	0.00 (−0.00. 0.00)	0.897	−0.03 (−0.32. 0.25)	0.804	0.00 (−0.00. 0.01)	0.167	−0.15 (−0.55. 0.25)	0.468	0.15 (−0.06. 0.36)	0.163
Allometric muscle power (W/m^2^)	0.09 (−4.88. 5.06)	0.971	−4.50 (−12.2. 3.27)	0.255	−0.01 (−0.12. 0.08)	0.756	−1.11 (−8.54. 6.20)	0.755	0.14 (−0.04. 0.33)	0.142	−3.46 (−13.73. 6.81)	0.507	4.07 (−1.67. 9.81)	0.163
Specific muscle power (W/kg)	0.00 (−0.04. 0.06)	0.766	−0.00 (−0.09. 0.08)	0.855	−0.008 (−0.09. 0.08)	0.855	−0.03 (−0.11. 0.05)	0.471	0.00 (−0.00. 0.00)	0.815	−0.00 (−0.01. 0.10)	0.873	0.09 (0.01. 0.175)	0.019


Analysis was adjusted for age, sex, body mass index, physical activity status, smoking status, any cardiovascular diagnosis, unintentional loss of weight, systolic blood pressure (except for SBP continuous), diastolic blood pressure (except for DBP continuous), and use of angiotensin converting enzyme inhibitors (except for ACEI-HTN). Abbreviations: 5STS, 5-time sit-to-stand; ACEI, angiotensin converting enzyme inhibitors; ACEI-HTN, hypertension treated with angiotensin converting enzyme inhibitors; ASM, appendicular skeletal muscle; BP-HTN, hypertension based on high systolic and diastolic blood pressure; CLI-HTN, medical diagnosis of hypertension; DPB, diastolic blood pressure; DBP-HTN, hypertension based on high diastolic blood pressure; IHG, isometric handgrip strength; SBP, systolic blood pressure, SBP-HTN, hypertension based on high systolic blood pressure; WS, walking speed

**Figure 1:**
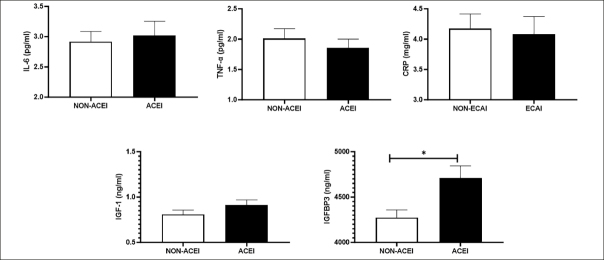
Blood concentrations of inflammatory markers, IGF-1, and IGFBP3 according to ACEI use Data are shown as mean ± standard deviation. *p<0.05. Abbreviations: Interleukin-6 levels, IL-6; Tumor necrosis factor-α, TNFα; ACEI, angiotensin-converting enzyme inhibitors; C-reactive protein, CRP; Insulin-like growth factor 1, IGF-1; IGF binding protein 3, IGFBP3.

Figure 1 shows blood concentrations of inflammatory markers, IGF-1, and IGFBP3 according to ACEI use. The use of ACEI was associated with higher levels of IGFBP3. No other significant differences were observed between groups.

**Figure 2:**
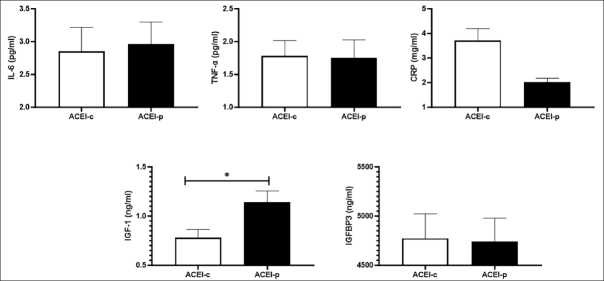
Blood concentrations of inflammatory markers, IGF-1, and IGFBP3 according to ACEI subtypes Data are shown as mean ± standard deviation. *p<0.05. Abbreviations: Interleukin-6 levels, IL-6; Tumor necrosis factor-α, TNFα; ACEI, angiotensin-converting enzyme inhibitors; C-reactive protein, CRP; Insulin-like growth factor 1, IGF-1; IGF binding protein 3, IGFBP3.

Comparisons for physical performance and ASM between ACEI-c and ACEI-p users are shown in Figure 2. Participants on ACEI-p had higher IGF-1 blood levels compared with ACEI-c users. No other significant differences were observed.

## Discussion

The main findings of the present study indicate that ACEI use might influence the association between hypertension-related parameters and ASM and physical performance in very old adults. Performance on the 5STS test was significantly and negatively associated with diastolic BP values. However, significance was lost when results were adjusted for ACEI use. Participants on ACEIs were more likely to have greater specific muscle power than non-ACEI users. When candidate molecules that could mediate the effects of ACEIs were examined, significance was found for IGF-1 and IGFBP3, with a possible influence of ACEI subtypes.

Our results are partly supported by other investigations that reported no significant associations between hypertension-related parameters and physical performance in older adults. Coelho-Junior et al. (10) found no significant associations between either hypertension or BP and balance, Timed “Up and Go!” (TUG), WS at normal and fast paces, IHG strength, 5STS, or lower-limb muscle power in community-dwelling older adults. Furthermore, Acar et al. (33) and Abate et al. (11) did not observe differences in dynamic or static balance tests between older adults with and without hypertension. Similar findings were reported in frail older adults (34).

Other investigations found that hypertensive individuals had worse physical performance than normotensive peers (8, 9). Results from longitudinal studies also showed detrimental effects of hypertension on physical performance. Rosano et al. (35) observed that hypertension accelerated WS decline in community-dwelling older adults with high baseline physical function over 18 years of follow-up. Laddu et al. (7) examined a large sample of postmenopausal women and found negative cross-sectional associations between 5STS and WS performances and systolic BP, while diastolic BP was positively correlated with IHG strength. A significant and positive association for IHG strength, 5STS, and WS with BP was observed in the longitudinal analysis. More recently, Kara et al. (36) conducted a multicentric observational study to examine associations between sarcopenia-related parameters and hypertension in apparently healthy Turkish middle-aged and older adults. After excluding participants with major clinical conditions, such as heart failure, stroke, and depression, authors found that the performance on IHG and 5STS tests in women, but not in men, was negatively associated with the presence of hypertension.

Discrepancies across studies might be due to sample characteristics (e.g., age, sex distribution), hypertension parameters (controlled vs. non-controlled), physical performance assessment tools (e.g., IHG vs. 5STS) (37), study design (e.g., case-control, cross-sectional, longitudinal), and statistical approach. Most studies did not adjust the analysis for potentially relevant covariates, including physical activity (38, 39), weight loss (40, 41), and the presence of comorbidities (e.g., diabetes, heart failure) (42). Furthermore, although pharmacological treatment was examined as a covariate in some studies, the merging of different drug classes into a single variable might mask the influence of specific medication types. In particular, ACEIs have been proposed as a possible pharmacological remedy against age-related neuromuscular decline. Onder et al. (43) observed that older adults on ACEIs had a milder decline in WS and upper-limb muscle strength over three years than those who used other antihypertensive drugs. Di Bari et al. (44) found greater lower extremity muscle mass in ACEI users than in those on other antihypertensive drugs. Spira et al. (24) and Bea et al. (45) reported similar results in participants of the Berlin Aging Study. Kara et al. (36) found that Turkish women on ACEIs had greater IHG strength but slower walking speed in comparison to those on other antihypertensive drugs. Though, other studies found no associations between ACEI use and neuromuscular parameters (23, 24, 25). Indeed, ACEI use was cross-sectionally associated with slower walking performance in older adults with hypertension (46).

In our study, we observed that the significant association between BP and 5STS performance was lost when the analysis was adjusted according to ACEI use. We also found that a high specific muscle power was more likely to be observed in participants on ACEIs. Moreover, higher blood IGFBP3 levels were found among ACEI users, whereas those on ACEI-p showed higher IGF-1 concentrations.

ANGII promotes muscle wasting by inhibiting anabolic pathways and triggering protein degradation (19). ANGII-induced muscle wasting is associated with a catabolic environment characterized by significant increases in oxidative stress markers, including NAD(P)H oxidase (14, 15, 16), superoxide dismutase (15, 47), and thiobarbituric acid reactive substances (14). ANGII also negatively affects mitochondrial biogenesis (16, 47) and function in muscle which translates into diminished ATP production (47) and increased oxidant generation (14, 15, 47). The impairment in mitochondrial bioenergetics shifts muscle fiber composition (14, 16) and impacts force development and exercise capacity (16). Notably, treatment with ACEIs ameliorates exercise capacity (14), improves mitochondrial respiration efficiency (14), decreases reactive oxygen species (ROS) production, and increases type I muscle fibers (14). ANGII promotes muscle protein breakdown through the activation of the ubiquitin-proteasome system (15, 16, 18, 19, 47). This pathway is partially dependent on the activation of caspases (15, 18), given that their blockade prevents ANGII-induced muscle wasting (18). At the same time, ANGII blocks the activation of AKT (15, 18, 47), reducing the expression of ser473 and p70 (15).

IGF-1 is a major regulator of muscle protein synthesis (48, 49, 50) by stimulating the AKT/mTOR pathway and inhibiting the activity of ubiquitin ligases (51, 52). The activity and bioavailability of IGF-1 are determined by its binding proteins, among which IGBP3 is the most abundant and the main carrier protein of IGF-1 in plasma (53, 54, 55). IGF-1 and IGFBP3 blood concentrations were found to be reduced in older adults with sarcopenia (54) and were significantly associated with muscle mass and IHG strength (53, 54, 56, 57).

An association between ACEIs and mediators of the IGF-1 pathway has been reported in literature. In preclinical models, ANGII-indued muscle atrophy was associated with reductions in circulating and muscular levels of IGF-1 and IGFBP3, whereas IGF-1 overexpression blunted the proteolytic effects of ANGII in muscle (16, 17, 18, 19). Treatment with captopril increased IGF-1, IGF-1 receptor, IGFBP3, and phosphorylated AKT expression in the rat myocardium (20, 21). In humans, Corbalan et al. (58) found that 8-week treatment with enalapril increased IGF-1 levels in adults with congestive heart failure. These findings were expanded by Giovannini et al. (22), who observed that adults with a high cardiovascular risk profile showed significant increases in IGF-1 and IGFBP3 blood levels after a 6-month treatment with fosinopril compared with placebo.

Taken together, these observations suggest that ACEI use may affect the interaction between physical function and hypertension-related parameters in very old adults. This effect is possibly mediated by the actions of ACEI on components of the IGF-1 pathway at peripheral level. A question that remains is why ACEIs affect the association between hypertension and specific lower limb muscle power, but not other physical performance tests. Although we do not have data to support our hypothesis, this scenario may be dependent on muscle mass. Indeed, specific muscle power was the only test adjusted by ASM values. The preservation of muscle mass is important not only for mechanical purposes, but also for endocrine activities (59, 60, 61). The contracting muscle synthetizes and secretes bioactive molecules called myokines, which include IGF-1 (62). Therefore, it is possible that ACEI use might have contributed to maintaining muscle mass and its endocrine function, thereby impacting IGF-1 levels observed in the subsample of participants on ACEI-p. Studies in experimental models of hypertension are needed to confirm these speculations.

The results of the present study may provide directions for future studies. Very old adults from rural areas on ACEIs might display greater specific muscle power, an important predictor of negative events, in comparison to non-ACEI users. These findings encourage the conduction of other observational studies, mainly examining very old adults, given that different mechanisms of BP regulation might be relevant to this population. In addition, investigations analyzing muscle power are scarce, since efforts to allow its field evaluation have grown only recently. Hence, this study might serve as a reference for future investigations. To the best of our knowledge, this is the second study to investigate the relationship between ACEI subtypes and physical performance. Our findings corroborate those by George et al. (21), who found no associations between ACEI subtypes and IHG or WS. However, ACEI-p have specific associations with the IGF-1 pathway. This property deserves further exploration.

Our study is not free of limitation. First, ASM was estimated according to calf circumference instead of recommended assessment tools (4). Second, blood concentrations of oxidative stress markers were not measured. Third, other aspects of ACEI treatment, such as dosage, duration, and adherence were not recorded, which might impact our results (23, 44). Fourth, the lack of a detailed description of the antihypertensive treatment (e.g., use of diuretics) prevented us from conducting deeper analyses. Fifth, the categorization of BP according to other criteria (e.g., JNC7 and JNC8) might change the study results. Sixth, the cross-sectional design of the study does not allow inference to be drawn on the time course of changes in the variables considered or on cause–effect relationships. Seventh, data on specific ACEI subtypes were not available for some participants. Finally, we examined a cohort of very old adults who lived in a mountain region, and extrapolations to other groups should be made with caution.

## Conclusions

Our results indicate that ACEI use might influence the association between hypertension-related parameters and ASM and physical performance in very old adults. Treatment with ACEIs may impact the association between BP and 5STS as reflected by the fact that older adults on ACEIs were more likely to have greater specific muscle power than non-ACEI users. This finding might be linked to the effects of ACEI with peripherical actions on the IGF-1 pathway.

*Contributions:* Data curation, HJCJ, RC, MT, AAB, FL, AP, EM.; Formal analysis, HJCJ, RC, AAB, AP, EM.; Investigation, HJCJ, RC, MT, AAB, FL, AP, EM.; Methodology, HJCJ, RC, MT, AAB, FL, AP, EM.; Writing–original draft, HJCJ, MT, AAB, FL; Writing–review & editing, HJCJ, RC, AP, EM.

*Funding:* This work was supported by the Università Cattolica del Sacro Cuore [D1.2020 and D1.2022], the Italian Ministry of Health [Ricerca Corrente 2023], and the nonprofit research foundation “Centro Studi Achille e Linda Lorenzon” [N/A]. The authors also acknowledge co-funding from Next Generation EU, in the context of the National Recovery and Resilience Plan, Investment PE8–Project Age-It: “Ageing Well in an Ageing Society”. This resource was co-financed by the Next Generation EU [DM 1557 11.10.2022]. The views and opinions expressed are only those of the authors and do not necessarily reflect those of the European Union or the European Commission. Neither the European Union nor the European Commission can be held responsible for them. Open access funding provided by Università Cattolica del Sacro Cuore within the CRUI-CARE Agreement.

*Conflicting of interest:* The authors declare that there is no conflict of interest.

*Ethics declarations:* The study was compliant with the principles of the Declaration of Helsinki. The protocol was approved by the Ethics Committee of the Università Cattolica del Sacro Cuore (Rome, Italy). All participants signed an informed consent prior to enrolment.

## Electronic Supplementary Material

**Supplementary table 1**. Unadjusted regression analysis for the association between hypertension-related parameters and physical performance.
