# Supplementary material for: Associations Between Hypertension, Angiotensin-Converting Enzyme Inhibitors, and Physical Performance in Very Old Adults: Results from the ilSIRENTE Study
Source: J Frailty Aging. 2024 Mar 6;13(2):74–81. doi: 10.14283/jfa.2024.15 (PMC12275770; doi:10.14283/jfa.2024.15)
Supplement: Supplementary file 1 — Supplementary table 1. Unadjusted regression analysis for the association between hypertension-related parameters and physical performance. [file mmc1.docx]

|  | **CLI-HTN** | | **SBP-HTN** | | **SBP continuous** | | **DBP-HTN** | | **DBP continuous** | | **BP-HTN** | | **ACEI-HTN** | |
| --- | --- | --- | --- | --- | --- | --- | --- | --- | --- | --- | --- | --- | --- | --- |
|  | **Adjusted β (95% CI)** | **p** | **Adjusted β (95% CI)** | **p** | **Adjusted β (95% CI)** | **p** | **Adjusted β (95% CI)** | **p** | **Adjusted β (95% CI)** | **p** | **Adjusted β (95% CI)** | **p** | **Adjusted β (95% CI)** | **p** |
| IHG (kg) | 0.87 (-2.2. 3.9) | 0.578 | 5.55 (2.27. 8.83) | 0.001 | 0.10 (0.04. 0.16) | 0.001 | 6.26 (3.06. 9.46) | 0.001 | 0.12 (0.01. 0.23) | 0.033 | 8.52 (4.89. 12.14) | 0.001 | 3.27 (-1.04. 7.59) | 0.136 |
| WS at normal pace (m/s) | -0.07 (-0.64. 0.04) | 0.799 | 0.02 (-0.03. 0.09) | 0.382 | 0.01 (0.00. 0.02) | 0.239 | 0.05 (-0.01. 0.11) | 0.116 | 0.00 (-0.00. 0.00) | 0.526 | 0.07 (0.00. 0.15) | 0.036 | 0.02 (-0.05. 0.10) | 0.466 |
| WS at fast pace (m/s) | -0.01 (-0.09. 0.06) | 0.711 | 0.07 (-0.01. 0.16) | 0.083 | 0.01 (0.00. 0.00) | 0.071 | 0.07 (-0.01. 0.16) | 0.085 | 0.00 (-0.00. 0.00) | 0.506 | 0.13 (0.03. 0.24) | 0.011 | 0.03 (-0.08. 0.14) | 0.604 |
| ASM (kg) | -0.34 (-1.47. 0.77) | 0.541 | 1.26 (0.06. 2.47) | 0.04 | 0.02 (0.00. 0.05) | 0.014 | 1.39 (0.20. 2.59) | 0.022 | 0.01 (-0.02. 0.05) | 0.521 | 2.08 (0.71. 3.45) | 0.003 | 1.02 (-0.54. 2.58) | 0.199 |
| 5STS (s) | 0.06 (-1.64. 1.77) | 0.94 | 0.49 (-1.49. 2.48) | 0.623 | -0.00 (-0.04. 0.02) | 0.672 | -1.22 (-3.16. 0.72) | 0.217 | -0.00 (-0.04. 0.02) | 0.672 | -0.96 (-3.47. 1.62) | 0.475 | -1.58 (-3.73. 0.56) | 0.148 |
| Absolute muscle power (W) | -3.84 (-19.1. 11.4) | 0.622 | -2.54 (-20.4. 15.3) | 0.779 | 0.01 (-0.31. 0.33) | 0.944 | 14.14 (-3.19. 31.4) | 0.109 | 0.20 (-0.39. 0.80) | 0.501 | 14.08 (-8.32. 36.50) | 0.217 | 21.27 (0.68. 41.86) | 0.04 |
| Relative muscle power (W/kg) | -0.05 (-0.27. 0.15) | 0.596 | -0.01 (-0.35. 0.15) | 0.434 | 0.01 (-0.31. 0.33) | 0.944 | 0.01 (-0.22. 0.26) | 0.889 | 0.00 (-0.00. 0.01) | 0.545 | -0.03 (-0.36. 0.29) | 0.85 | 0.25 (0.00. 0.50) | 0.049 |
| Allometric muscle power (W/m^2^) | -1.68 (-7.29. 3.92) | 0.554 | -1.57 (-8.11. 4.96) | 0.636 | 0.01 (-0.31. 0.33) | 0.944 | 5.15 (-1.18. 11.5) | 0.111 | 0.08 (-0.13. 0.30) | 0.423 | 4.11 (-4.30. 12.5) | 0.336 | 5.41 (-1.37. 12.2) | 0.117 |
| Specific muscle power (W/kg) | -0.01 (-0.07. 0.05) | 0.775 | -0.00 (-0.08. 0.07) | 0.955 | 0.00 (-0.00. 0.00) | 0.6 | 0.00 (-0.08. 0.07) | 0.872 | 0.00 (-0.00. 0.00) | 0.749 | 0.02 (-0.07. 0.12) | 0.642 | 0.11 (0.01. 0.22) | 0.021 |

**Supplementary table 1.** Unadjusted regression analysis for the association between hypertension-related parameters and physical performance.

Abbreviations: 5STS, 5-time sit-to-stand; ACEI, angiotensin converting enzyme inhibitors; ACEI-HTN, hypertension treated with angiotensin converting enzyme inhibitors; ASM, appendicular skeletal muscle; BP-HTN, hypertension based on high systolic and diastolic blood pressure; CLI-HTN, medical diagnosis of hypertension; DPB, diastolic blood pressure; DBP-HTN, hypertension based on high diastolic blood pressure; IHG, isometric handgrip strength; SBP, systolic blood pressure, SBP-HTN, hypertension based on high systolic blood pressure; WS, walking speed.
